# tDCS-induced episodic memory enhancement and its association with functional network coupling in older adults

**DOI:** 10.1038/s41598-019-38630-7

**Published:** 2019-02-19

**Authors:** Daria Antonenko, Dayana Hayek, Justus Netzband, Ulrike Grittner, Agnes Flöel

**Affiliations:** 1 Charité – Universitätsmedizin Berlin, corporate member of Freie Universität Berlin, Humboldt-Universität zu Berlin, and Berlin Institute of Health, Department of Neurology, NeuroCure Clinical Research Center, Charitéplatz 1, 10117 Berlin, Germany; 2 Department of Neurology, Universitätsmedizin Greifswald, Ferdinand-Sauerbruch-Straße, Greifswald, Germany; 3 Berlin Institute of Health (BIH), Anna-Louisa-Karsch-Straße 2, 10178 Berlin, Germany; 4 Charité – Universitätsmedizin Berlin, corporate member of Freie Universität Berlin, Humboldt-Universität zu Berlin, and Berlin Institute of Health, Institute of Biometry and Clinical Epidemiology, Charitéplatz 1, 10117 Berlin, Germany; 5 Charité – Universitätsmedizin Berlin, corporate member of Freie Universität Berlin, Humboldt-Universität zu Berlin, and Berlin Institute of Health, Center for Stroke Research, Charitéplatz 1, 10117 Berlin, Germany

## Abstract

Transcranial direct current stimulation (tDCS) augments training-induced cognitive gains, an issue of particular relevance in the aging population. However, negative outcomes have been reported as well, and few studies so far have evaluated the impact of tDCS on episodic memory formation in elderly cohorts. The heterogeneity of previous findings highlights the importance of elucidating neuronal underpinnings of tDCS-induced modulations, and of determining individual predictors of a positive response. In the present study, we aimed to modulate episodic memory formation in 34 older adults with anodal tDCS (1 mA, 20 min) over left temporoparietal cortex. Participants were asked to learn novel associations between pictures and pseudowords, and episodic memory performance was subsequently assessed during immediate retrieval. Prior to experimental sessions, participants underwent resting-state functional magnetic resonance imaging. tDCS led to better retrieval performance and augmented learning curves. Hippocampo-temporoparietal functional connectivity was positively related to initial memory performance, and was positively associated with the magnitude of individual tDCS-induced enhancement. In sum, we provide evidence for brain stimulation-induced plasticity of episodic memory processes in older adults, corroborating and extending previous findings. Our results demonstrate that intrinsic network coupling may determine individual responsiveness to brain stimulation, and thus help to further explain variability of tDCS responsiveness in older adults.

## Introduction

Research aiming at the facilitation and augmentation of cognitive processes through non-invasive brain stimulation (NIBS) in the course of aging is an area of great current interest^1,2^. In particular, transcranial direct current stimulation (tDCS) has been suggested to tune ongoing network processes^3–5^, to increase inter-regional functional communication and to reverse age-related network reorganization^6^. Beyond its potential for cognitive enhancement, tDCS may also reveal the magnitude of preserved neuroplasticity in older adults^7–9^.

Recently, several studies have reported an age-dependency of neural and behavioral effects of tDCS^7,10–12^. For instance, Fiori and colleagues showed that verbal learning during temporoparietal anodal tDCS was enhanced in older but not young adults^12^. Martin and colleagues observed comparable behavioral impact on semantic word fluency induced by tDCS in older and young adults, but found differential task-related network modulation^11^. Other studies could not corroborate beneficial effects of tDCS on memory performance in older adults^13,14^. Based on evidence for age-related structural deterioration and associated functional brain-wide network reorganization, interventional techniques may operate upon other neural processes than in young brains^1^. Importantly, age-related cortical changes not only affect the magnitude of tDCS-induced modulation but also the pattern of underlying network reorganization^10–12,15^. Beside the general variability in tDCS effects^16^, responsiveness may even vary more among older adults due to large inter-individual differences in age-related deterioration of cognitive performance and brain structure^8,17^, most likely explaining differential effects of tDCS in young versus older adults. What seems indisputable thus far is that results from young adults regarding efficient stimulation parameters as well as expected interactions with underlying regional brain activity might not be transferable to older brains^1,17^. In sum, despite the large neuroscientific interest, the understanding of tDCS effects on the aged brain is still incomplete. The heterogeneity of findings further highlights the complexity of underlying mechanisms.

Among cognitive domains, episodic memory processes exhibit the most crucial age-related impairment^18^, thus representing a core target for interventional strategies such as tDCS. At the same time, these processes are difficult to target directly as they are mainly mediated by medial temporal brain structures, such as the hippocampus^19^. However, activity in these brain areas may be modulated by stimulating functionally connected cortical regions, as suggested by both functional magnetic resonance imaging (fMRI) studies^19–21^ and previous NIBS studies, using transcranial magnetic stimulation^22,23^ and tDCS with temporoparietal stimulation targets^12,15,24–26^. In addition, modulation of verbal episodic memory formation has been demonstrated by anodal tDCS over the left prefrontal cortex^14,27–30^. These findings confirm the involvement of a widespread neural network of medial temporal, temporoparietal and frontal areas in episodic memory processes, with several nodes of the network being susceptible to modulation with brain stimulation^19,31^. Further, tDCS experiments have highlighted a laterality-dependent benefit for episodic memory in older adults, with right temporoparietal stimulation improving memory for visuospatial^15,24^ and left temporoparietal stimulation for verbal information^12,27^. No study to date investigated the relationship of individual tDCS-induced episodic memory enhancement with intrinsic network coupling in older adults.

In the present study, we aimed to investigate these open issues, using anodal tDCS over left temporoparietal cortex to modulate episodic memory formation. Specifically, we hypothesized that tDCS would enhance memory as assessed by retrieval performance after picture-word associative learning. In addition, we expected that tDCS would lead to steeper learning curves over multiple blocks. Further, we acquired resting-state functional images to examine the relationship individual memory network coupling with episodic memory formation at baseline as well as with the magnitude of tDCS-induced enhancement. Here, we hypothesized that individual memory network coupling would be positively associated with memory formation and tDCS-induced memory enhancement.

## Results

### tDCS-induced learning and memory improvement

An episodic memory task was administered that required participants to learn picture-pseudoword associations during five learning blocks with concurrent tDCS application (1 mA, 20 min). Subsequent retrieval was assessed in two “transfer” blocks (one immediate, one with a 20-min delay) where previously presented pictures were replaced by corresponding words and participants had to identify correct pairs. Performance on immediate retrieval was defined as main outcome. To examine the effects of tDCS on memory performance and learning curves, percentage of correct responses and reaction times for retrieval and learning blocks were subjected to linear mixed model analyses with stimulation condition (sham, anodal) as within-subject factor. Models were adjusted for age and the order of experimental sessions.

#### Memory performance

**Figure 1:**
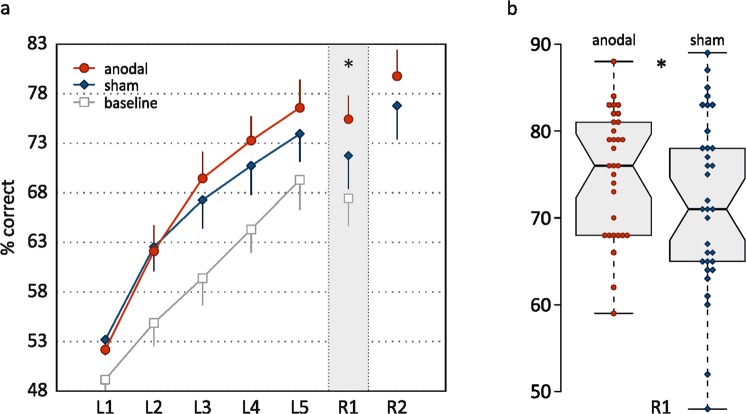
Accuracy in the episodic memory task. (a) Percentage of correct responses in the five learning blocks (L1-L5) and the two retrieval blocks (R1, R2). Means and (one side of) two-sided 95%-CIs are shown. (b) Boxplots and individual data points for main outcome variable R1, created with BoxPlotR (http://boxplot.tyerslab.com/)^72^. *p < 0.05.

To address the question whether memory performance differed significantly between stimulation conditions after learning (primary endpoint), performance during the immediate retrieval block was compared. Performance after anodal stimulation was on average 2.8% better compared to sham in the 1^st^ retrieval block (β = 2.8, 95%-CI: [0.3, 5.4], F_(1,89)_ = 4.815, *p* = 0.031; linear mixed model post-hoc, N = 34, 127 data points; Fig. 1). The main effect of stimulation condition on memory performance indicated superior performance in the anodal compared to the sham stimulation condition of on average 2.3% (β = 2.3, 95%-CI: [0.5, 4.2], F_(1,91)_ = 6.30, *p* = 0.014). There were no condition by retrieval block interaction (β = −1, 95%-CI: [−4.6, 2.6], F_(1,90)_ = 0.30, *p* = 0.585) or condition by age interaction effects (β = 0.01, 95%-CI: [−0.25, 0.27], F_(1,93)_ = 0.01, *p* = 0.927). Performance on the 2^nd^ retrieval block was on average 4.6% better compared to the 1^st^ (β = 5.1, 95%-CI: [2.8, 6.4], F_(1,89)_ = 25.58, *p* < 0.001). Effect of age was not statistically significant (β = −0.3, 95%-CI: [−0.6, 0.1], F_(1,33)_ = 2.43, *p* = 0.129). A session effect revealed practice effects over the experimental sessions with an average improvement of 2.9% (β = 0.029, 95%-CI: [1.1, 4.8], F_(1,91)_ = 9.70, *p* = 0.002).

There was no difference between stimulation conditions in reaction times (main effect: β = −1.2, 95%-CI: [−20.3, 17.9], F_(1,90)_ = 0.02, *p* = 0.899). Reaction time on the 2^nd^ retrieval block was shorter compared to the 1^st^ (main effect: β = −29.6, 95%-CI: [−55.2, −4.0], F_(1,88)_ = 11.05, *p* = 0.001). Effect of age was not significant (β = 0.9, 95%-CI: [−2.5, 4.2], F_(1,32)_ = 0.02, *p* = 0.899). A session effect revealed faster responses on the second experimental session (β = 25.0, 95%-CI: [6.0, 44.1], F_(1,91)_ = 6.80, *p* = 0.011). Interactions between condition and retrieval block (β = −2.6, 95%-CI: [−39.5, 34.3], F_(1,89)_ = 0.02, *p* = 0.889) and condition and age (β = −1.4, 95%-CI: [−4.0, 1.3], F_(1,93)_ = 1.06, *p* = 0.307) were non-significant.

#### Learning performance

Performance accuracy during five learning blocks was analyzed using a linear mixed model with blocks as level-one units nested in different individuals who were level-two units, in order to test for differences in the learning curves between stimulation conditions. The effect of stimulation condition itself was not significant (main effect: β = 0.4, 95%CI: [−0.8, 1.6] F_(1,287)_ = 0.52, *p* = 0.473; linear mixed model, N = 34, 320 data points; Fig. 1), but the interaction of condition and learning block indicated steeper learning curves in the anodal compared to the sham stimulation condition (β = 1.0, 95%-CI: [0.2, 1.8], F_(1,281)_ = 6.16, *p* = 0.014). Overall, task performance improved over the learning blocks; improvement showed a curvilinear convex shape indicated by a linear increase of approximately 5% per block (β for the five learning blocks [centered and linear] = 4.9, 95%-CI: [4.4, 5.5], F_(1,281)_ = 687.61, *p* < 0.001) and an additional negative coefficient for the square of block order (β [squared] = −1.1, 95%-CI: [−1.4, −0.7], F_(1,281)_ = 38.00, *p* < 0.001). The negative age effect revealed flatter learning curves with higher age (main effect: β = −0.2, 95%-CI: [−0.5, 0.0], F_(1,33)_ = 4.68, *p* = 0.038). A session effect indicated practice effects over the experimental sessions of 2.5% (β = 2.5, 95%-CI: [1.3, 3.7], F_(1,287)_ = 16.32, *p* < 0.001). There was no interaction effect of condition and age (β = −0.02, 95%-CI: [−0.19, 0.15] F_(1,294)_ = 0.05, *p* = 0.827).

**Figure 2:**
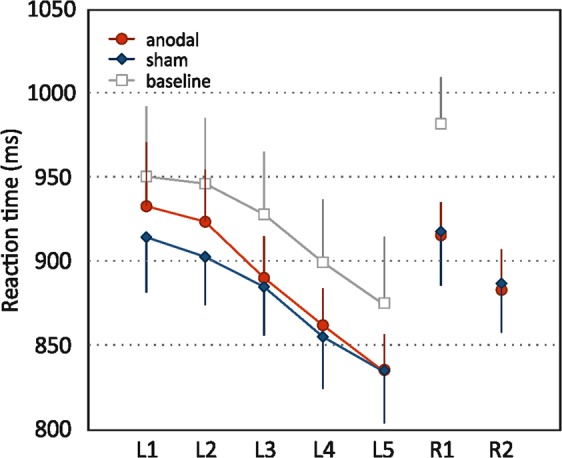
Reaction time in the memory task. Mean reaction time (in ms) during the five learning blocks (L1-L5) and the two retrieval blocks (R1, R2). Means and (one side of) two-sided 95%-CIs are shown.

Reaction time became shorter over the course of learning blocks (β = −21.1, 95%-CI: [−27.0,−15.2], F_(1,279)_ = 123.20, *p* < 0.001; Fig. 2) as well as experimental sessions (β = −28.3, 95%-CI: [−40.8,−15.8], F_(1,284)_ = 19.80, *p* < 0.001). An age effect on reaction time indicated slower responses with higher age, but was not statistically significant (β = 3.2, 95%-CI: [0.2, 6.2], F_(1,32)_ = 3.70, *p* = 0.063). Effect of stimulation condition (β = 7.5, 95%-CI: [−5.0, 20.1], F_(1,284)_ = 1.40, *p* = 0.237), condition by learning block interaction (β = −6.0, 95%-CI: [−14.5, 2.6] F_(1,281)_ = 1.90, *p* = 0.169), and condition by age interaction (β = −1.0, 95%-CI: [−2.8, 0.7] F_(1,290)_ = 1.29, *p* = 0.258) were not statistically significant.

### Functional network correlates

#### Baseline correlations

**Figure 3:**
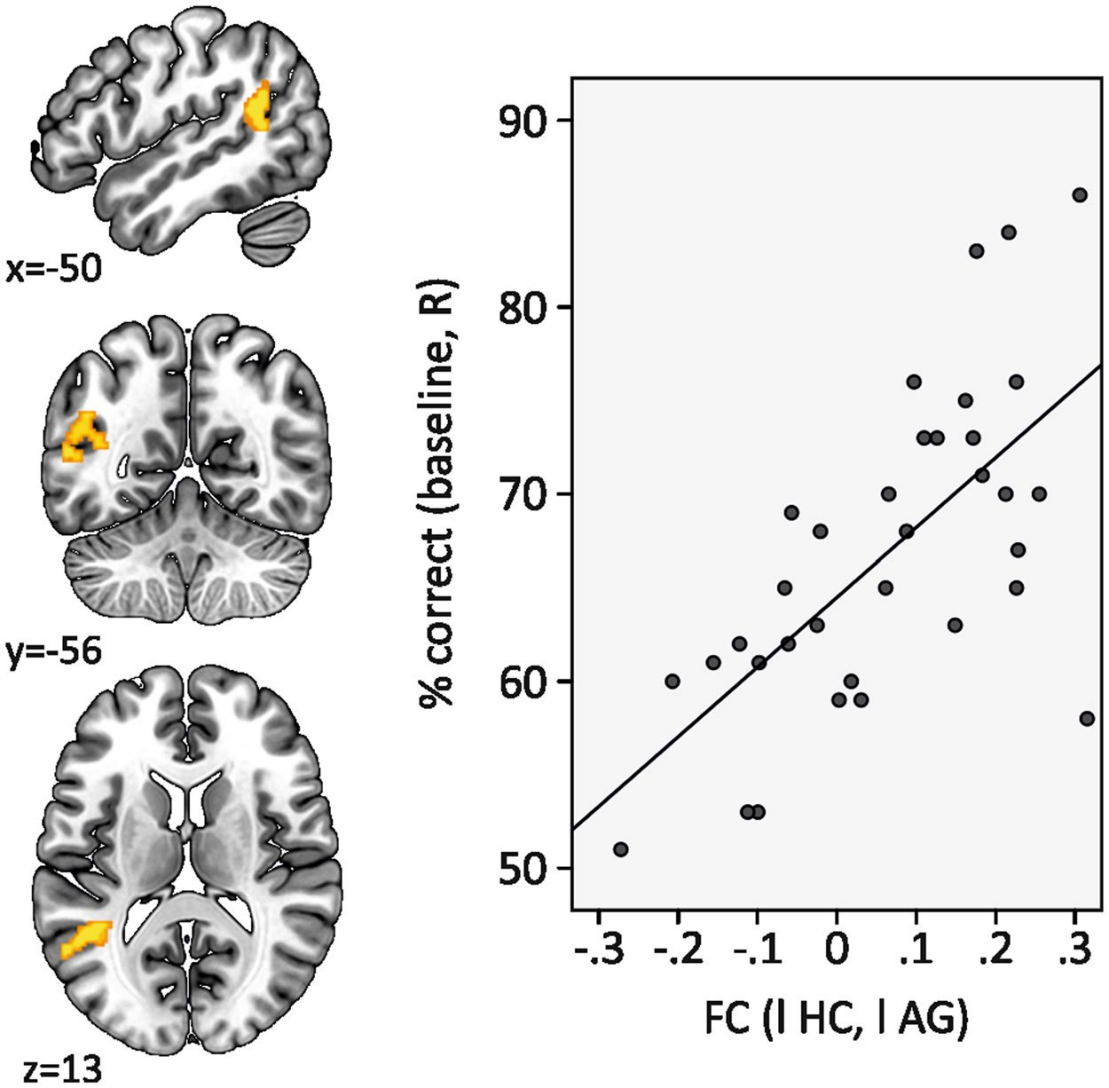
Scatterplot of functional connectivity and memory performance. Functional connectivity between the left hippocampus and left temporoparietal cortex correlated with task performance at baseline (R, retrieval). The significant cluster that emerged from whole-brain seed-to-voxel analyses had peak coordinates of x = −40, y = −52, z = 18, T = 4.45, cluster size *k* = 232 mm², cluster-*p*-FDR = 0.040. The scatterplot illustrates the relationship (baseline performance is plotted over individual functional connectivity that was extracted from the significant cluster).

To determine whether functional coupling within the memory network at rest predicted memory performance at baseline, seed-to-voxel correlation maps were calculated for the left hippocampus (i.e., Pearson’s r correlation of the blood-oxygenation level-dependent (BOLD) time course of the hippocampus with all other brain voxels). Subsequent general linear model analysis for the dependent variable baseline memory performance revealed an association of task performance with left-hemisphere hippocampo-temporoparietal coupling (significant cluster in left angular gyrus, peak coordinates: x = −40, y = −52, z = 18, T = 4.45, *k* = 232 voxel, cluster-*p*-FDR = 0.040, cluster-*p*-unc < 0.001, Fig. 3, corrected for age).

#### Association of functional connectivity with individual tDCS-induced improvement

**Figure 4:**
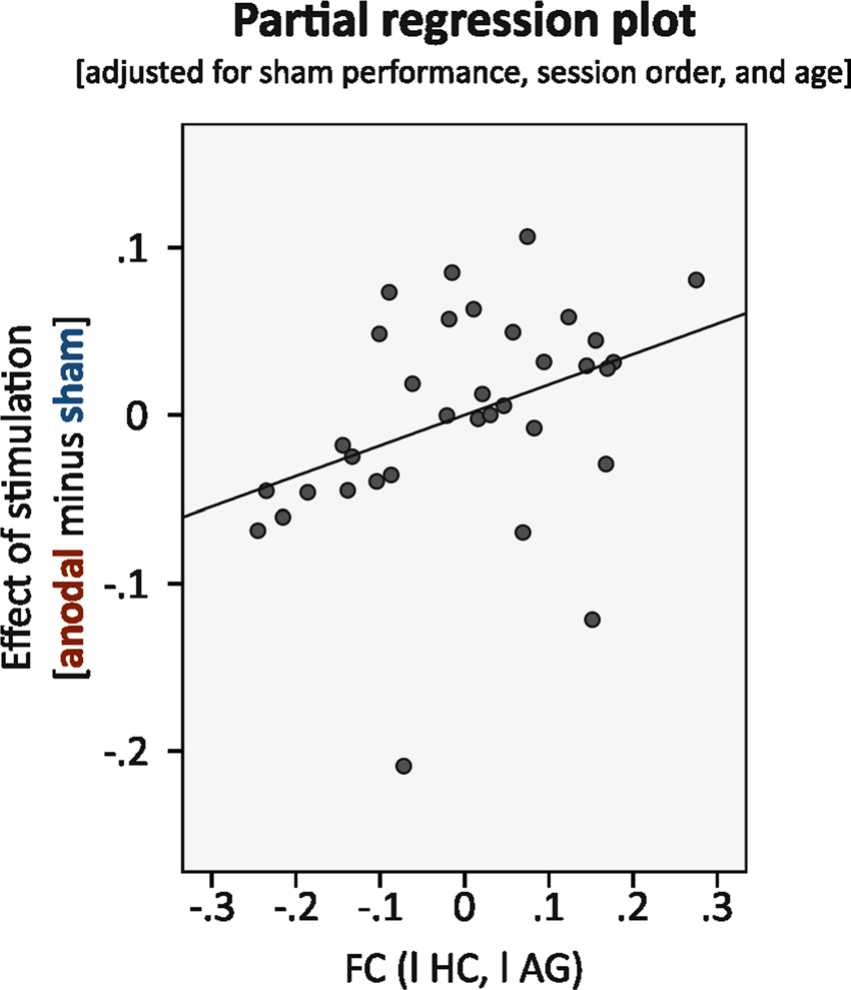
Partial regression plot (residuals) for the association between functional connectivity and individual responsiveness to tDCS (β = 0.31, p = 0.036). Linear regression included the difference in performance in anodal and sham stimulation condition as dependent variable and functional connectivity between hippocampus and angular gyrus as independent variable, adjusted for performance in sham stimulation condition, session order and age.

We then aimed to investigate whether the individual left-hemisphere hippocampo-temporoparietal coupling at baseline was also associated with the magnitude of responsiveness to tDCS. The latter was defined as difference in memory performance between anodal and sham stimulation (“percentage of correct responses during immediate retrieval in atDCS” minus “percentage of correct responses during immediate retrieval in stDCS”). Pearson’s r correlation coefficients between BOLD time series in the left hippocampus and in the significant cluster in the left angular gyrus were extracted to obtain individual memory network coupling. We performed a linear regression adjusted for sham stimulation performance, session order and age. Sham performance was included as a covariate in the statistical model because we wanted to account for “performance without stimulation” in the evaluation of individual improvement through anodal stimulation. We found that individual responsiveness to tDCS was positively associated to functional connectivity (standardized β = 0.31, p = 0.036; see Table 1 for all model coefficients, overall model R² = 0.58, adjusted R² = 0.52; Fig. 4).

**Table 1:** Multiple linear regression analysis for difference in memory performance (anodal minus sham) (dependent variable) (n = 34).

Independent variables	B	SE	Standardized β	t	*p*
Constant	0.626	0.187		3.251	0.002
Sham performance	−0.706	0.140	−0.826	−5.044	0.000
Session order	−0.004	0.028	−0.022	−0.142	0.888
Functional connectivity	0.182	0.083	0.305	2.195	*0*.*036*
Age	−0.002	0.002	−0.135	−1.035	0.309

Note. R² = 0.58, adjusted R² = 0.52.

### Control variables, mood ratings and adverse effect questionnaire

In order to control for effects of attention or working memory capacity, we conducted digit span forward and backward both before and after experimental sessions (Table 2). Forward and backward digit span did not differ between stimulation conditions (forward: β = 0.06, 95%-CI: [−0.38, 0.50], F_(1,100)_ = 0.07, *p* = 0.790; backward: β = 0.1, 95%-CI: [−0.38, 0.47], F_(1,100)_ = 0.04, *p* = 0.836) and time points (forward: β = 0.21, 95%-CI: [−0.23, 0.64], F_(1,100)_ = 0.88, *p* = 0.351; backward: β = −0.22, 95%-CI: [–0.64, 0.20], F_(1,100)_ = 1.08, *p* = 0.302; linear mixed models; N = 34, 136 data points).

**Table 2:** Digit span test performance.

	Anodal stimulation	Sham stimulation
Forward		
Pre	7.85 (2.12)	7.91 (2.07)
Post	7.79 (2.26)	7.59 (2.21)
Backward		
Pre	7.12 (2.21)	7.00 (2.30)
Post	7.24 (2.17)	7.34 (2.09)

Mean (SD) values.

Positive and negative affect ratings before and after the experimental sessions are shown in Table 3. Positive, and negative affect did not differ between stimulation conditions (positive: β = −0.05, 95%-CI: [−0.17, 0.07], F_(1,100)_ = 0.60, *p* = 0.440; negative: β < 0.01, 95%-CI: [−0.02, 0.03], F_(1,100)_ = 0.03, *p* = 0.855) and time points (positive: β = 0.10, 95%-CI: [−0.02, 0.22], F_(1,100)_ = 2.87, *p* = 0.093; negative: β = −0.01, 95%-CI: [−0.04, 0.02], F_(1,100)_ = 0.54, *p* = 0.466; linear mixed models; N = 34, 136 data points).

**Table 3:** Mood ratings.

	Anodal stimulation	Sham stimulation
Positive affect		
Pre	3.31 (0.82)	3.32 (0.84)
Post	3.27 (0.81)	3.16 (0.92)
Negative affect		
Pre	1.07 (0.11)	1.07 (0.10)
Post	1.08 (0.12)	1.09 (0.14)

Mean (SD) values.

Number of participants who reported the respective adverse effect are shown in Table 4. Tingling during stimulation was most commonly reported in both stimulation conditions (in total by 47% of the participants). However, the occurrence of all adverse effects did not differ between conditions (all chi-square’s ≤ 3.1, all *p*’s ≥ 0.08).

**Table 4:** Adverse effect ratings after the last experimental session.

	Anodal stimulation	Sham stimulation
Pain	3	1
Tingling	11	5
Itchiness	2	1
Burning	2	1
Fatigue	2	3
Tension	3	4
Loss of concentration	5	5
Headache	0	0
Discomfort	1	0

Number of participants. Total N = 34.

## Discussion

The present study investigated tDCS-induced modulation of episodic memory processes in older adults as well as the relationship with functional network coupling on an individual level. Memory performance was enhanced after anodal tDCS over left temporoparietal cortex, i.e., participants retrieved significantly more correct pairings of newly acquired picture-pseudoword associations in the transfer task. Additionally, older participants exhibited steeper learning curves during anodal compared to sham stimulation. Functional memory network coupling between left hippocampus and left temporoparietal brain area was positively associated with the magnitude of individual tDCS-induced cognitive enhancement.

Previous studies that used anodal tDCS over the left temporoparietal cortex to modulate associative learning and memory have reported benefits for performance of young adults^25,26,32^. For example, Flöel *et al*. found accelerated learning and improved retrieval of newly acquired picture-word pairs when learning was accompanied by anodal tDCS, using identical task and stimulation parameters^26^. In the present study, older adults likewise exhibited faster learning success during stimulation compared to sham and enhanced subsequent memory. Thus, our findings extend previous results from young adults to the aging population, and indicate preserved responsiveness of episodic memory networks in older adults. Anodal tDCS over left temporoparietal cortex could have enhanced memory formation by modulating synchronous activity or connectivity within the memory network comprising temporoparietal and hippocampal structures^22,26,33^. In older adults, beneficial effects for verbal learning of anodal tDCS over left temporoparietal cortex were supported by Fiori *et al*. who further suggested that simultaneous cathodal tDCS over right homologue may even be superior to a unilateral electrode montage^12,34^. Modulation of episodic memory functions has been also shown with prefrontal stimulation targets in older adults^27–30^. Anodal tDCS over left dorsolateral prefrontal cortex enhanced memory for words and reduced forgetting when applied during the encoding^30^, reconsolidation^29^ or recall phase^27^. A recent study showed that also ventrolateral prefrontal tDCS during intentional encoding enhanced delayed recognition memory in older adults^28^. These findings corroborate the hypothesis that hippocampus-dependent memory processes are mediated by the inter-regional interaction within a distributed network in older adults^19,31^, and are susceptible to modulation through lateral frontal and parietal brain stimulation^28,29,35^. In sum, the group-level comparison between anodal temporoparietal and sham stimulation conditions in our study corroborated and extended previous findings by demonstrating preserved plasticity of episodic memory processes in older adults.

As we observed beneficial effects on both learning curves and subsequent retrieval, the question whether memory enhancement in our study was caused primarily by modulation of encoding, of consolidation, or of retrieval processes remains open. However, from previous reports, there is more evidence favoring the hypothesis that tDCS preferentially enhances post-encoding processes, such as consolidation and retrieval of acquired material^30,36–38^. Offline effects may thus be stronger than online effects and of particular relevance as they may also persist after a period of consolidation^14,38–40^. In sum, it is currently unclear whether initial encoding or retrieval processes, or both, are modulated with temporoparietal tDCS, and further studies are needed to approach this question^41^.

Recent studies have highlighted the need to report and examine individual data in tDCS studies in order to better understand inter-subject variability in responsiveness and its neurobiological correlates^42–44^. Given the complex mechanisms underlying tDCS effects, including interacting state-dependent brain activity, task demands, stimulation parameters and a set of inter-individual factors, it seems not surprising that findings between studies are heterogeneous and also depend on the study sample^4^. In older cohorts, the topic may even be more relevant as, in addition to variability in behavioral performance, variability of the rate of structural decline in aging likely also affects individual sensitivity to plasticity-inducing mechanisms^8^. As in Brosnan *et al*., we observed a large range of variability in individual responsiveness to tDCS within our sample of older adults. As sources of variability still need to be determined, detailed report of sample characteristics, individual factors and its correlative relationships within the sample under study will help to further advance our understanding of underlying mechanisms^42–44^.

By showing a positive correlation between memory performance and intrinsic hippocampo-temporoparietal functional coupling, our data further support the hypothesis that successful memory formation may be dependent on the connection between posterior inferior parietal and medial temporal brain regions^19,45^. Thus, in our group of older adults, variability in task performance may be predicted by individual intrinsic connectivity within the memory network. This finding corroborates previous evidence for the crucial role of hippocampal-cortical networks for memory functions^37,46,47^. Our finding further emphasizes the importance of anatomical connection, and coordinated activity, between medial temporal and parietal areas for successful memory retrieval in older adults^47^. This link would then allow to modulate hippocampus-dependent processes by targeting connected cortical sites^22,41,48^.

Further, we assessed whole-brain hippocampal functional connectivity to evaluate if individual tDCS responsiveness was predicted by baseline functional network coupling in older adults. We found that hippocampo-temporoparietal coupling was positively associated with the magnitude of individual tDCS-induced memory enhancement. This finding is in line with prior research suggesting a pivotal role of intrinsic hippocampal coupling in age-related memory decline^49^. Specific connections, including synchronous activity between hippocampus and angular gyrus may constitute central mechanisms underlying functional decline. Modulation of synchronous activity within and between networks appears not only to be crucial in the course of brain aging^50^, but has been also suggested as main underlying mechanisms of tDCS effects^51,52^. Our data supports the notion that functional connectivity at rest can be used as a predictor for individual response to brain stimulation^41,53^ and that posterior brain areas may be viable target regions for neuromodulatory techniques in the context of age-related deficits^8^. Here, the combination of brain stimulation and neuroimaging to study neuromodulatory effects during and after tDCS will have to further elucidate its complex interaction with brain activity and age-related modulation of memory processes^54,55^.

The present study does not allow drawing firm conclusions about spatial specificity of the applied electrode configuration due to the large size of the temporoparietal electrode as well as the lack of testing other electrode configurations. Conventional dual-electrode tDCS set-ups stimulate networks rather than brain regions^2,34,52^. In particular, the targeted temporoparietal region has a heterogeneous organization with regard to its cytoarchitectonic, connectional and functional diversity^45,56^, playing a role in several higher-order cognitive processes in humans^45^. Therefore, it is conceivable that other brain networks not primarily involved in memory processes were stimulated as well. Our computational modeling result confirms that maximal electric field strengths are distributed over the left lateral temporal and parietal areas, with high intensities around the intended target. Please note though that spatial specificity of tDCS is thought to be increased by concurrent task-related activity, indicating that the interaction of brain stimulation with a task induces more focused effects^57,58^. Moreover, the correlative functional connectivity analysis suggested that intrinsic synchronous activity in hippocampus and angular gyrus may be involved in successful memory performance in older adults. Thus, even though we cannot draw firm conclusions about spatial specificity, our set-up demonstrated tDCS-induced tuning of ongoing memory network processes and preserved plasticity in older adults^3,8,9^. Further, our data does not allow concluding whether the observed effects are specific to older adults, as no young group was included. However, we believe that at this point, detailed examination of older cohorts is paramount, to better understand variability in this group^1^.

Prospectively, persistence of benefits is essential to promote the development of clinical applications in the context of age-related diseases and may be achieved with combined tDCS-training interventions^15,59^. Future studies should therefore evaluate additional biomarkers determining individual responsiveness^60^ and probe the efficiency of tDCS to produce sustained plasticity induction in older adults, for which our current mechanistic study provides important groundwork.

## Materials and Methods

### Participants and experimental procedure

Thirty-four healthy older adults participated in the study (16 female, min/max age: 51/80 years, see Table 5 for participant characteristics). All were native German speakers, were right-handed, and had no history of neurological or psychiatric disorders. Neuropsychological testing was administered prior to study inclusion in order to assure normal cognitive functioning (CERAD-Plus, http://memoryclinic.ch). Performances on all subtests were not below 1.5 SD’s according to age- and education-related normative scores. The study was approved by the ethics committee of the Charité University Medicine and conducted in accordance with the Helsinki Declaration. Written informed consent was obtained from all participants prior to participation.

**Table 5:** Sample characteristics.

	Mean	SD
Age, years	63.1	7.7
Education, years	15.3	2.3
LQ^a^	93.5	9.8
GDS^b^	1.3	1.4
Digit Span (max. 14)		
Forward	7.5	2.3
Backward	6.0	1.8
Vocabulary test (max. 37)^c^	33.3	2.0
Semantic fluency, *N* (in 60 *s*)	25.3	5.7
Boston Naming Test, *N* (max. 15)	14.6	0.5
Mini-Mental State (max. 30)	29.4	0.8
Word list learning, *N*		
Total (max. 30)	23.3	3.2
Trial 1 (max. 10)	6.2	1.5
Trial 2 (max. 10)	8.1	1.3
Trial 3 (max. 10)	9.0	1.0
Word list retrieval, *N* (max. 10)	8.3	1.2
Word list intrusions, *N*	0.9	1.9
Figure copying, *N* (max. 11)	11.0	0.0
Figure retrieval, *N* (max. 11)	10.7	0.7
Phonemic fluency, *N* (in 60 *s*)	16.1	4.1
Trail making test, *s*		
Part A	39.8	11.6
Part B	83.8	32.5

^a^LQ, laterality quotient^73^.

^b^GDS, Geriatric Depression Scale^74^.

^c^^75^.

Older adults participated in three experimental sessions. The first session (baseline) included MRI scanning (acquisition of resting-state data) and subsequent administration of the episodic memory task. In the two subsequent experimental sessions, tDCS was applied (either anodal or sham in counterbalanced order, separated by one week) over the left temporoparietal cortex during the learning phase of the task.

### tDCS

A battery-driven stimulator (neuroConn DC-Stimulator Plus; neuroCare Group GmbH, Munich, Germany) was used to deliver direct current to the scalp via two sponge-electrodes soaked into saline solution. The anodal electrode (5 × 7 cm²) was placed centrally over CP5 according to the 10–10 EEG system, while the cathode (10 × 10 cm²) was placed over the right supraorbital area (centered over the right anterior frontal cortex, or AF4, respectively). The larger size of the sponge (10 × 10 cm²) has been shown to reduce current density to a level (0.01 mA/cm²) that does not exert functional neurophysiological effects^61^. Due to these results, it is assumed that the effects underneath the cathode are functionally less efficient (but not “inert”)^62^. Episodic memory processes as assessed in our study have been shown to be mediated by distributed networks including medial temporal areas such as the hippocampus^20^ and lateral temporoparietal areas^19–21,59^. As anodal tDCS over the left temporoparietal cortex has been shown to modulate performance in similar tasks as the one used in our study^25,26,32^, it was chosen as target area for stimulation. The software SimNIBS 2.1.1 was used to estimate the electric field induced by tDCS, based on the finite element method and individualized tetrahedral head meshes generated from T1- and T2-weighted structural MR images of one healthy older participant (http://simnibs.org; see Supplementary Information for details on current field modeling)^63,64^. Modeling results showed that maximal electric field strengths are distributed over left lateral temporal and parietal areas, with high intensities around the intended target area, i.e., in the vicinity of the left angular gyrus (Figure S1). Electrodes were fixed with two rubber bands and impedance was kept below 5 kOhm. Duration of anodal stimulation was 20 min (starting with the first learning block of the task, covering the first four learning blocks of the task) whereas in sham stimulation current was turned off after 30 sec. Stimulation intensity was 1 mA with 10 sec fade in and fade out.

Before and after each stimulation condition, positive and negative affect schedule (PANAS) was administered. Participants had to rate their current mood on 10 positive and 10 negative items (on a Likert scale with a range of 1 to 5)^65^. Change of ratings between time points was compared between conditions. After the completion of the last experimental session, participants were asked to retrospectively report the occurrence of adverse effects during the last session in a standardized questionnaire^66^.

### Episodic memory task

The episodic memory paradigm was adapted from previous studies^20,26,67,68^ and programmed using the software Presentation (Neurobehavioral Systems, http://www.neurobs.com/, version 18.1). The paradigm consists of the presentation of pseudoword-picture pairs. For each participant, a set of 30 pseudowords and 30 pictures of daily life objects were randomly matched to 30 “correct” pairings to create different correct (and, therefore, also incorrect) pseudoword-picture combinations.

Three different sets of stimuli (A, B, C) were used for three sessions (baseline, sham, anodal), containing different pseudowords and pictures^26^. The order of the set presentation was counterbalanced across participants. In each session, five learning blocks followed by an immediate retrieval block were presented. In the stimulation conditions (sham, anodal), an additional retrieval block was administered after a delay of 20 min.

During the learning blocks, a total of 600 trials were presented (120 trials per block). “Correct” pairings occurred ten times in total (i.e., twice in each block). In addition, each of the 30 pictures was presented ten times with varying “incorrect” pseudowords (i.e., two different “incorrect” pairings for each picture per block). Each “incorrect” pairing was presented only once over the course of learning. The order of trial presentation was randomized. Participants were not informed about the underlying frequency principle, and were instructed to decide as quickly as possible if word and picture match. Thus, the learning principle of this associative learning phase involves higher co-occurrences of “correct” arbitrary couplings compared to “incorrect” couplings (ratio 10:1)^68^. In each learning trial, the picture was presented 200 ms after the onset of the auditory spoken pseudoword (delivered over headphones) and remained on the screen for 1500 ms^67^. During picture presentation, participants had to decide whether the pairing was “correct” or “incorrect” by pressing one of two response buttons (using their left or right index finger, respectively).

During retrieval blocks, learning success was measured in a “transfer” task. Here, instead of presenting a picture, corresponding German spoken words were delivered with the pseudowords. Stimulus count, the underlying frequency principle (i.e., two “correct” and two “incorrect” pairings per block), and trial timings were identical to those in the learning phase. Percentage of correct responses and mean reaction time of each block were assessed. Main outcome measure for memory performance was percentage of correct responses during the immediate retrieval block^26^.

### MRI acquisition

Brain imaging data were acquired at the Berlin Center of Advanced Neuroimaging using a 3T Siemens Trio Scanner with a standard 12-channel head coil. A three-dimensional structural scanning protocol was applied using high-resolution T1-weighted magnetization-prepared rapid gradient echo imaging (1 × 1 × 1 mm³ voxel size; flip angle = 9 deg, repetition time = 1900 ms, echo time = 2.52 ms, 192 slices). Acquisition of resting-state fMRI was performed using an echo-planar imaging sequence (3 × 3 × 4 mm³ voxel size; flip angle = 78 deg, repetition time = 2070 ms, echo time = 30 ms, descending acquisition, 172 volumes). Participants were instructed to keep their eyes closed, not to fall asleep or think of anything in particular. None of the participants fell asleep during the scanning interval as per self-report. An additional fluid attenuated inversion recovery sequence was acquired for neuroradiological assessment and to exclude structural abnormalities.

### fMRI analysis

Functional connectivity analysis was performed using the CONN toolbox version 17f (www.nitrc.org/projects/conn, RRID:SCR_009550)^69,70^. We used the default data preprocessing pipeline provided by the toolbox which includes functional realignment, slice-time correction, structural segmentation and normalization to the Montreal Neurological Institute (MNI) template, functional segmentation and normalization, and smoothing (with 6-mm Gaussian kernel). The realignment confound (derived from the estimated motion parameters) was defined by 6 rigid-body dimensions plus 6 first-order temporal derivatives using Artifact Detection Toolbox (ART, www.nitrc.org/projects/artifact_detect/). Confounds in the blood oxygenation level-dependent (BOLD) signal from physiological and other spurious sources of noise were estimated and regressed out using the anatomical CompCor method implemented in the toolbox^71^. Anatomical images were segmented into grey matter (GM), white matter (WM), and cerebrospinal fluid (CSF) masks using SPM12. To minimize partial voluming with GM the WM and CSF masks were eroded by one voxel^70^. Functional data were temporally filtered (at high-pass threshold 0.01 Hz). First-level (within-subjects) seed-to-voxel correlation maps were calculated for the left hippocampus (i.e., Pearson’s r between the residual BOLD time-course of the seed mask and the time course of all other voxels in the brain were computed).

For second-level (between-subjects) general linear model analyses, correlation coefficients were transformed to normally distributed z scores (using Fisher’s transformation). To examine the association of hippocampal connectivity with baseline task performance, first-level connectivity maps for each participants were entered into a whole-brain regression analysis with age as covariate. The reported cluster survived a height threshold of uncorrected *p* < 0.005 (positive contrast) and an extent threshold of FDR-corrected *p* < 0.05 at the cluster level. To obtain individual ROI-to-ROI connectivity values of hippocampal-temporoparietal coupling, Pearson’s r correlation coefficients between BOLD time series in the left hippocampus and in the significant cluster in the angular gyrus were extracted.

### Statistical analysis

Statistical analyses were conducted using IBM SPSS Statistics 24 (http://www-01.ibm.com/software/uk/analytics/spss/). Linear mixed models (random intercept models) were computed for dependent variables with the factor stimulation condition (anodal, sham). Models were adjusted for age and experimental session. Linear trends were tested with an index variable (centered) for task blocks (i.e., five for learning and two for retrieval). For learning, an additional quadratic term for learning blocks was entered into the model. Linear regression analysis was conducted for association between functional connectivity and individual responsiveness to tDCS (defined by performance in anodal minus performance in sham stimulation condition), adjusted for age, order of experimental sessions and sham stimulation performance. Logistic regression models were computed for the comparison of adverse event occurrences. No corrections for multiple comparisons were applied. A two-sided significance level of α = 0.05 was used.

## Supplementary information

Supplementary Information

## Data Availability

The datasets generated during the current study are available from the corresponding author on reasonable request.
