# Supplementary material for: tDCS-induced episodic memory enhancement and its association with functional network coupling in older adults
Source: Sci Rep. 2019 Feb 19;9:2273. doi: 10.1038/s41598-019-38630-7 (PMC6381175; doi:10.1038/s41598-019-38630-7)
Supplement: Supplementary file 1 — Supplementary Information [file 41598_2019_38630_MOESM1_ESM.pdf]

Supplementary Information

"tDCS-induced episodic memory enhancement and its association with functional network coupling in older adults"

Daria Antonenko[1,2*], Dayana Hayek[1,2], Justus Netzband[1], Ulrike Grittner[3,4,5], Agnes Flöel[1,2,5*]

[1] Charité – Universitätsmedizin Berlin, corporate member of Freie Universität Berlin, Humboldt-Universität zu Berlin, and Berlin Institute of Health, Department of Neurology, NeuroCure Clinical Research Center, Charitéplatz 1, 10117 Berlin, Germany; [2] Department of Neurology, University of Greifswald, Greifswald, Germany; [3] Berlin Institute of Health (BIH), Anna-Louisa-Karsch 2, 10178 Berlin, Germany [4] Charité – Universitätsmedizin Berlin, corporate member of Freie Universität Berlin, Humboldt-Universität zu Berlin, and Berlin Institute of Health, Institute of Biometry and Clinical Epidemiology, Charitéplatz 1, 10117 Berlin, Germany, [5] Charité – Universitätsmedizin Berlin, corporate member of Freie Universität Berlin, Humboldt-Universität zu Berlin, and Berlin Institute of Health, Center for Stroke Research, Charitéplatz 1, 10117 Berlin, Germany

Electric field simulation

We acquired a structural MRI dataset of an older adult (m, 67 years) to model the applied stimulation protocol. The dataset was acquired on a 3 T Siemens Verio equipped with a 32-channel head coil at the Baltic Imaging Center. High-resolution T1w- (1 x 1 x 1 mm^3, TR = 2300 ms, TE = 2.96 ms, TI = 900 ms, flip angle = 9°; using selective water excitation for fat suppression) and T2w-weighted images (1 x 1 x 1 mm^3, TR = 12770 ms, TE = 86 ms, flip angle = 111°) were recorded.

The software SimNIBS 2.1.1 was used to estimate the electric field induced by tDCS, based on the finite element method and individualized tetrahedral head meshes generated from the structural MR images of the participant (http://simnibs.org) [1,2]. Head reconstruction was performed using the incorporated headreco tool based on SPM12 and CAT12 toolboxes [3]. Electrode positions were placed using the individual EEG coordinates, calculated based on four fiducials (Iz, Nz, LPA, RPA) in order to obtain the 10/10 EEG positions [4]. The anode was placed over CP5 (current: 1 mA), the cathode (current: -1 mA) was placed over the right anterior frontal cortex centered over AF4. Electrode parameters were modeled as follows: Electrode sizes of 5 x 7 cm^2, sponge sizes of 5 x 7 cm^2 (anode) and 10 x 10 cm^2 (cathode), electrode thickness of 2 mm, sponge thickness of 8 mm, connector sizes of 1 x 2 cm, and connector positions at the posterior (anode), and superior (cathode) edge of the electrodes. Default conductivity values provided by SimNIBS were used (σ(white matter)=0.126 S/m, σ(grey matter)=0.275 S/m, σ(cerebrospinal fluid)=1.654 S/m, σ(bone)=0.010 S/m, σ(scalp)=0.465) [5]. Simulation results (norm E) were interpolated to the individual cortical surface.

Figure S1 shows the results of electric field modeling. Maximal electric field strengths are distributed over left-lateral temporal and parietal areas, with high intensities around the intended target area, i.e., in the vicinity of the left temporoparietal cortex, or angular gyrus, respectively.

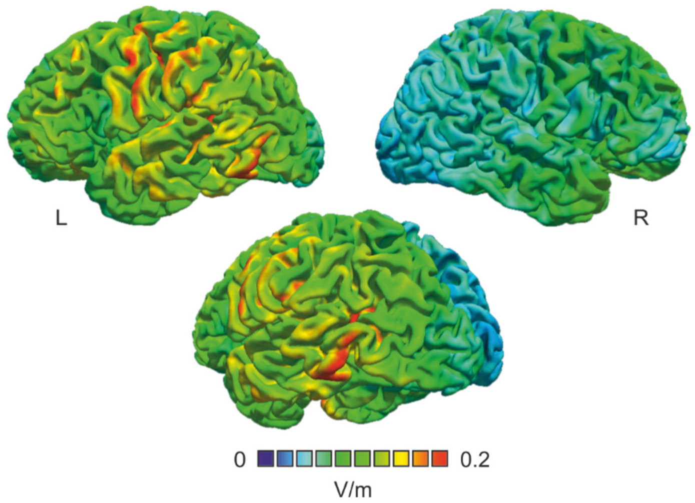

Figure S1. *Current field modeling for the stimulation configuration used in our study: anode centered over CP5 (7 x 5 cm²), cathode centered over AF4 (10 x 10 cm²), derived from FEM calculations using SimNIBS. Simulations results are shown for one older subject (m, 67 years), interpolated on the individual cortical surface. The distribution of electric field strength |E| is shown.*

1 Thielscher, A., Antunes, A. & Saturnino, G. B. Field modeling for transcranial magnetic stimulation: A useful tool to understand the physiological effects of TMS? *Conference proceedings : ... Annual International Conference of the IEEE Engineering in Medicine and Biology Society. IEEE Engineering in Medicine and Biology Society. Annual Conference* **2015**, 222-225, doi:10.1109/embc.2015.7318340 (2015).

2 Windhoff, M., Opitz, A. & Thielscher, A. Electric field calculations in brain stimulation based on finite elements: an optimized processing pipeline for the generation and usage of accurate individual head models. *Human brain mapping* **34**, 923-935, doi:10.1002/hbm.21479 (2013).

3 Nielsen, J. D. *et al.* Automatic skull segmentation from MR images for realistic volume conductor models of the head: Assessment of the state-of-the-art. *NeuroImage* **174**, 587-598, doi:10.1016/j.neuroimage.2018.03.001 (2018).

4 Jurcak, V., Tsuzuki, D. & Dan, I. 10/20, 10/10, and 10/5 systems revisited: their validity as relative head-surface-based positioning systems. *NeuroImage* **34**, 1600-1611, doi:10.1016/j.neuroimage.2006.09.024 (2007).

5 Thielscher, A., Opitz, A. & Windhoff, M. Impact of the gyral geometry on the electric field induced by transcranial magnetic stimulation. *NeuroImage* **54**, 234-243, doi:https://doi.org/10.1016/j.neuroimage.2010.07.061 (2011).
